# Supplementary material for: Complexome profiling of the Chlamydomonas psb28 mutant reveals TEF5 as an early PSII assembly factor
Source: Plant Cell. 2025 Mar 17;37(6):koaf055. doi: 10.1093/plcell/koaf055 (PMC12164590; doi:10.1093/plcell/koaf055)
Supplement: koaf055_Supplementary_Data [file koaf055_supplementary_data.zip › psb28_interactive.zip!/plots/intensity/qualityPlots/peptides/_Cre10.g457650.t1.2.html]

### Cre10.g457650.t1.2

Trivial name:   
SeqCoverage [median]: 11.00  
Mass: 15.00 kDa  
  
 

| Sequence | MissedCleavage | Mass [Da] | wtIntensity | muIntensity | Score |
| --- | --- | --- | --- | --- | --- |
| ASVDDEVLPTDEIAK | 0 | 1600.78 | 3035.31 | 2425.42 | 168.02 |
|  |  |  | 3035.3 | 2425.4 |  |
